# Feasibility of determining external beam radiotherapy dose using LuSy dosimeter

**DOI:** 10.1002/acm2.14387

**Published:** 2024-05-22

**Authors:** Janatul Madinah Wahabi, Jeannie Hsiu Ding Wong, Ghafour A. Mahdiraji, Ngie Min Ung

**Affiliations:** ^1^ Department of Biomedical Imaging Faculty of Medicine Universiti Malaya Kuala Lumpur Malaysia; ^2^ Radiotherapy and Oncology Department National Cancer Institute Putrajaya Malaysia; ^3^ Universiti Malaya Research Imaging Centre (UMRIC), Faculty of Medicine Universiti Malaya Kuala Lumpur Malaysia; ^4^ Flexilicate Sdn. Bhd. Universiti Malaya Kuala Lumpur Malaysia; ^5^ Clinical Oncology Unit Faculty of Medicine Universiti Malaya Kuala Lumpur Malaysia

**Keywords:** conformal radiotherapy, IMRT, plastic scintillator, SRS, VMAT

## Abstract

**Introduction:**

Radiation dose measurement is an essential part of radiotherapy to verify the correct delivery of doses to patients and ensure patient safety. Recent advancements in radiotherapy technology have highlighted the need for fast and precise dosimeters. Technologies like FLASH radiotherapy and magnetic‐resonance linear accelerators (MR‐LINAC) demand dosimeters that can meet their unique requirements. One promising solution is the plastic scintillator‐based dosimeter with high spatial resolution and real‐time dose output. This study explores the feasibility of using the LuSy dosimeter, an in‐house developed plastic scintillator dosimeter for dose verification across various radiotherapy techniques, including conformal radiotherapy (CRT), intensity‐modulated radiation therapy (IMRT), volumetric‐modulated arc therapy (VMAT), and stereotactic radiosurgery (SRS).

**Materials and methods:**

A new dosimetry system, comprising a new plastic scintillator as the sensing material, was developed and characterized for radiotherapy beams. Treatment plans were created for conformal radiotherapy, IMRT, VMAT, and SRS and delivered to a phantom. LuSy dosimeter was used to measure the delivered dose for each plan on the surface of the phantom and inside the target volumes. Then, LuSy measurements were compared against an ionization chamber, MOSFET dosimeter, radiochromic films, and dose calculated using the treatment planning system (TPS).

**Results:**

For CRT, surface dose measurement by LuSy dosimeter showed a deviation of ‐5.5% and ‐5.4% for breast and abdomen treatment from the TPS, respectively. When measuring inside the target volume for IMRT, VMAT, and SRS, the LuSy dosimeter produced a mean deviation of ‐3.0% from the TPS. Surface dose measurement resulted in higher TPS discrepancies where the deviations for IMRT, VMAT, and SRS were ‐2.0%, ‐19.5%, and 16.1%, respectively.

**Conclusion:**

The LuSy dosimeter was feasible for measuring radiotherapy doses for various treatment techniques. Treatment delivery verification enables early error detection, allowing for safe treatment delivery for radiotherapy patients.

## 1 INTRODUCTION

Radiotherapy aims to deliver a high dose of ionizing radiation to the tumor while maintaining a low dose to the surrounding normal tissues. Ionizing radiation is delivered using a medical linear accelerator with various photon and electron energies. Point dose or relative dose profile measurements are part of the quality assurance (QA) activities in radiotherapy.^1^ Dose measurements can be performed before or during the treatment procedure to verify if the dose delivered is the same as those predicted by the treatment planning system (TPS). It is often necessary to carry out pre‐treatment QC tests for advanced treatment techniques, such as intensity‐modulated radiotherapy (IMRT), volumetric‐modulated arc therapy (VMAT), and stereotactic radiosurgery (SRS). These techniques utilize complex movement of the multileaf collimator (MLC), gantry movement, and high dose per fraction.

The ionization chamber is recognized as the standard dosimeter because it is an absolute dosimeter with traceability to the standard laboratory.^1^ In addition, an ionization chamber is often used as a reference dosimeter when comparing and calibrating other relative dosimeters. However, because of the relatively large chamber sizes, these chambers may not be suitable for in vivo dose measurements. For the past few decades, the development of plastic scintillator dosimeters has enabled them to become a suitable dosimeter for radiotherapy. Plastic scintillator dosimeters can be constructed of tissue‐equivalent properties, fast decay time, high temporal and spatial resolution, and real‐time signal acquisition.^2^

Systematic validation of plastic scintillator dosimeters was first demonstrated by Beddar et al. in 1992.^3^, ^4^ They characterized their plastic scintillator dosimeter and measured basic beam data in radiotherapy, such as the percentage depth dose (PDD). From then on, research on a scintillation dosimeter had shown a steady rise with a focus on the scintillator material,^5^, ^6^ development of the photodetector,^7^, ^8^, ^9^ innovation on the optical guide,^10^, ^11^ elimination of the stem effect,^12^, ^13^, ^14^ and the dimension of measurements.^15^, ^16^ Plastic scintillator dosimeters have been used to tackle complex and advanced dosimetry challenges in radiotherapy, such as the small field,^16^ particle therapy,^17^ magnetic influence,^18^ and ultra‐high dose rate.^19^ These works demonstrated the promising potential of plastic scintillators in radiotherapy.

In this study, we evaluate the feasibility of the radioLUminescent dosimetry SYstem (LuSy) measuring doses for conformal radiotherapy, IMRT, VMAT, and SRS. LuSy dosimeter is a plastic scintillator dosimeter that utilizes SP101 plastic scintillator as the sensing material. LuSy dosimeter was first characterized and used to measure low‐energy x‐rays in mammography.^20^ Subsequently, it was characterized in high‐energy x‐rays.^21^ To date, no studies have investigated the feasibility of SP101 plastic scintillator in measuring clinical radiotherapy treatment beams with various treatment techniques. This work will contribute to the ongoing effort of others in plastic scintillation dosimetry.

## 2 MATERIALS AND METHOD

### 2.1 Radiation dosimeters

**FIGURE 1:**
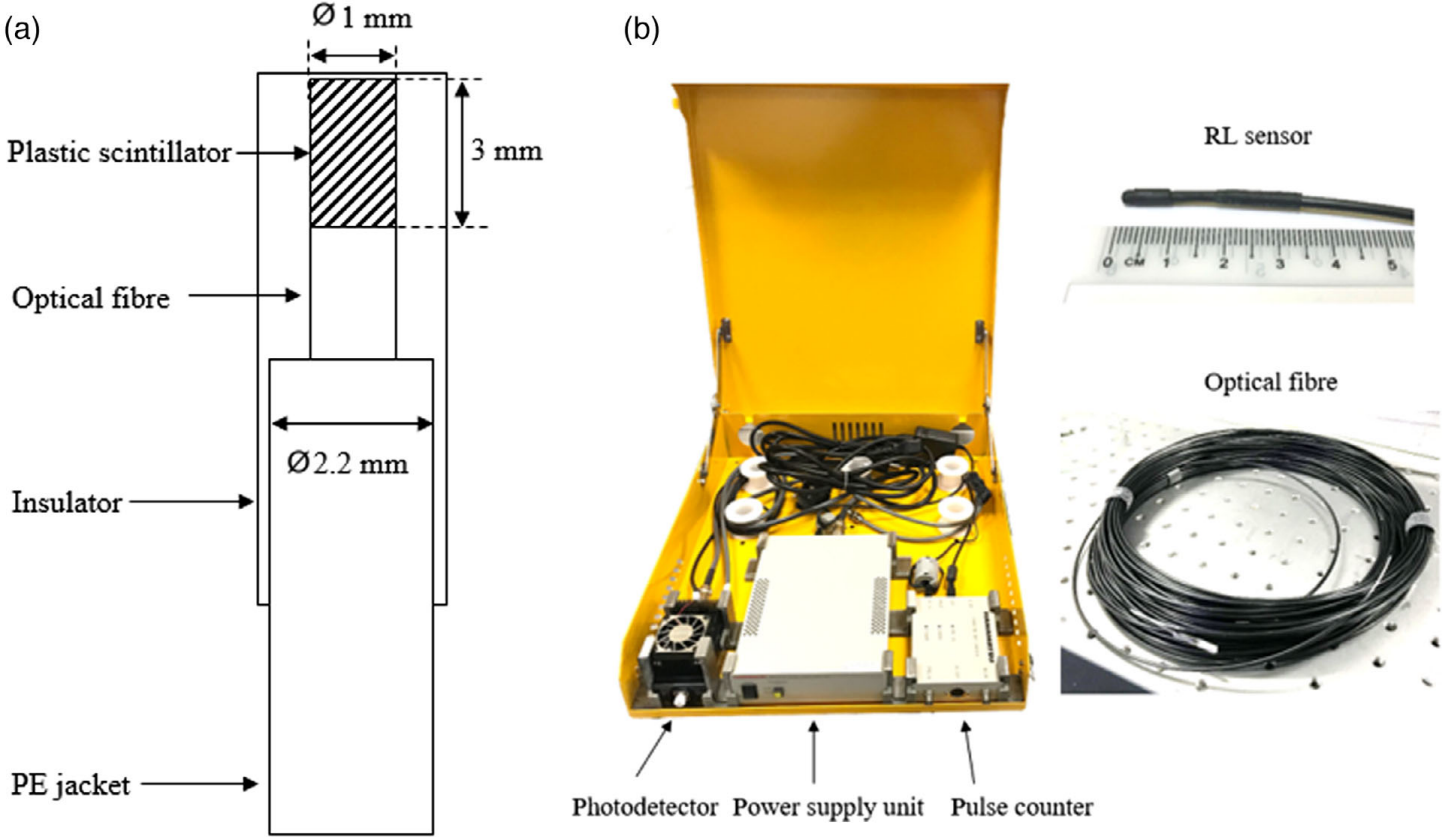
(a) Schematic diagram of the RL sensor and (b) the LuSy dosimeter.

LuSy is a plastic scintillator‐based radiation dosimetry system developed in‐house. It comprises an SP101 (Shalom EO, Hangzhou, China) plastic scintillator with a geometrical dimension of 1 mm diameter and 3 mm length (Figure 1a). The SP101 plastic scintillator has an effective atomic number of 6.58. Preliminary characterization of the LuSy dosimeter reported the dosimeter to be dose rate independent, with a slight over‐response of 6.5% for 10 MV relative to the 6 MV photon beams.^21^

The plastic scintillator was coupled to a 1 mm diameter PMMA optical fiber. Before coupling, the ends of the plastic scintillator and the optical fiber were polished using 1 µm grain size polishing paper. This assembly is called the radioluminescent (RL) sensor, and it was covered using the heat‐shrink tube for ambient light insulation. The optical fiber was 20 m long, carrying the light generated by the plastic scintillator to the photodetector (Hamamatsu Photonics KK, Shizuoka, Japan) (Figure 1b). The counted signal was recorded using LabVIEW (National Instruments, Tokyo, Japan) software and converted into a dose using an in‐house Matlab (The Mathworks Inc., Massachusetts, USA) script.

**FIGURE 2:**
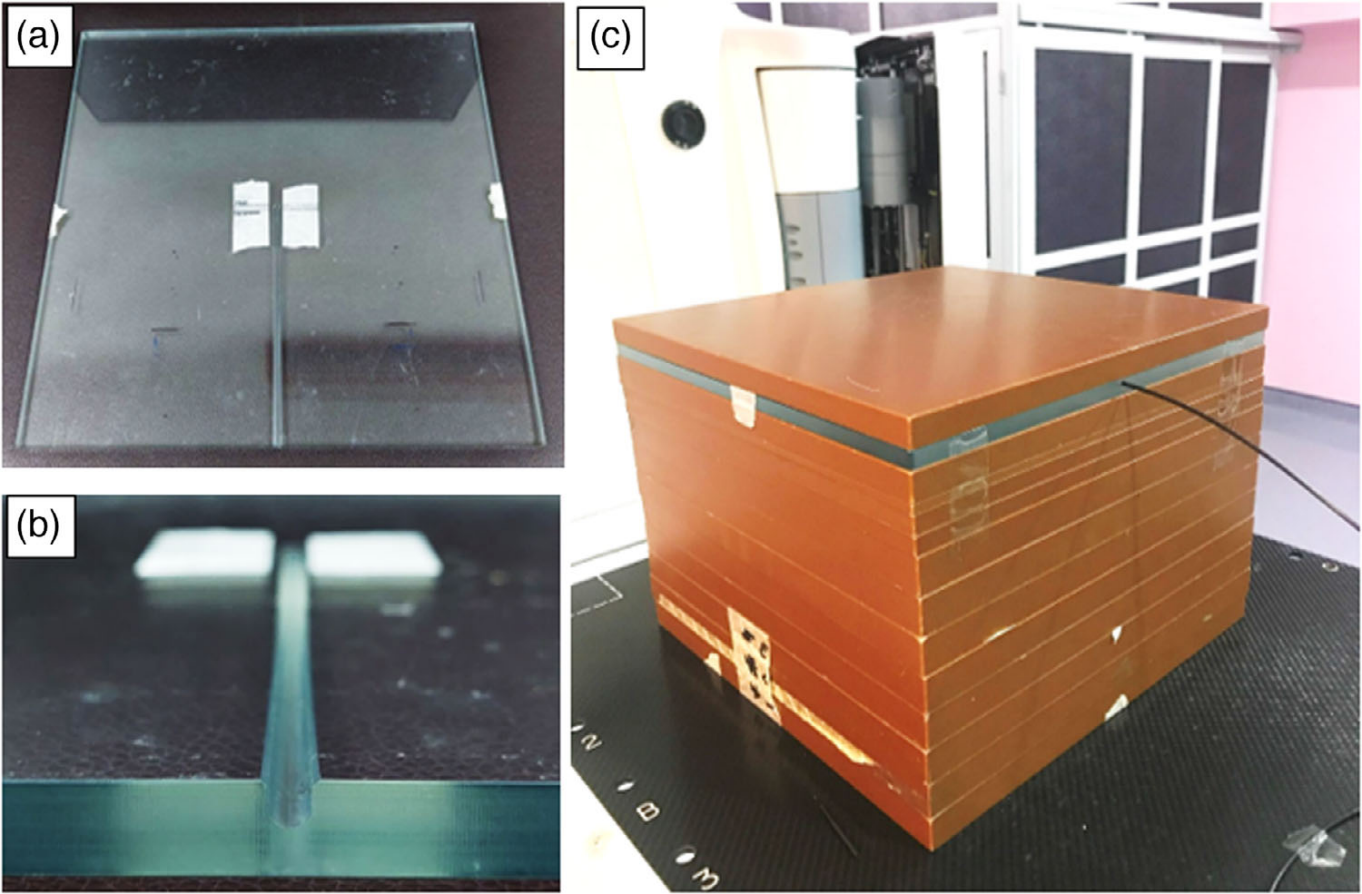
The fabricated acrylic phantom for Lusy. (a) The top view showing the length of the center groove, (b) the frontal view showing the shape and depth of the groove, and (c) the calibration setup with solid water phantom as the build‐up and backscatter material.

LuSy was calibrated using a 6 MV photon beam at a depth of maximum dose. The LuSy sensor was placed on a fabricated 30 × 30 cm^2^ acrylic phantom with 1 cm thickness (Figure 2). The solid water phantom was used as a build‐up and backscatter material. The acrylic phantom had a center groove to fix the position of LuSy sensor during dose measurement. The field size was 10 × 10 cm^2^, while the source‐to‐surface distance was 100 cm. The delivered dose ranged between 5 cGy and 500 cGy at a 600 MU/min dose rate. The resulting calibration coefficient was 244.64 counts/cGy.

For comparison, an ionization chamber, Gafchromic EBT3 film and a MOSFET‐based dosimeter were used in this study. The CC13 ionization chamber (IBA Dosimetry GmbH, Schwarzenbruck, Germany) has 0.13 cm^3^ volume, a standard chamber used for QC activities in radiotherapy. The CC13 was calibrated by the local secondary standard dosimetry laboratory (SSDL). Gafchromic EBT3 film (Ashland ISP, New Jersey, USA) is a radiochromic film composed of a 27 µm thick active layer sandwiched with symmetrical polyester layers and emulsion layers, resulting in a total thickness of 0.28 mm.^22^ The Gafchromic EBT3 is suitable for surface dose measurement in radiotherapy due to its thin geometry. The MO*Skin* detector (developed and prototyped by the Center for Medical Radiation Physics, University of Wollongong) is a MOSFET‐based dosimeter, and it has a 0.07 mm water equivalent depth (WED) in tissue, which makes it suitable for surface dose measurements.^23^

### 2.2 Radiation facilities and experimental setup

A Novalis Tx linear accelerator (LINAC) (Varian Medical System, California, USA and BrainLAB AG, Feldkirchen, Germany) equipped with a 120 high‐definition multileaf collimator (120HD MLC) was used as the radiation source. The MLC is arranged in opposed pairs where the 64 central MLC has a 2.5 mm width and the rest of the MLC with 5 mm width. The LINAC was calibrated according to IAEA TRS398, where 1 MU delivered equals 1 cGy at a reference condition (10 × 10 cm^2^ field size and 100 cm source‐to‐surface distance).

### 2.3 Treatment planning

**FIGURE 3:**
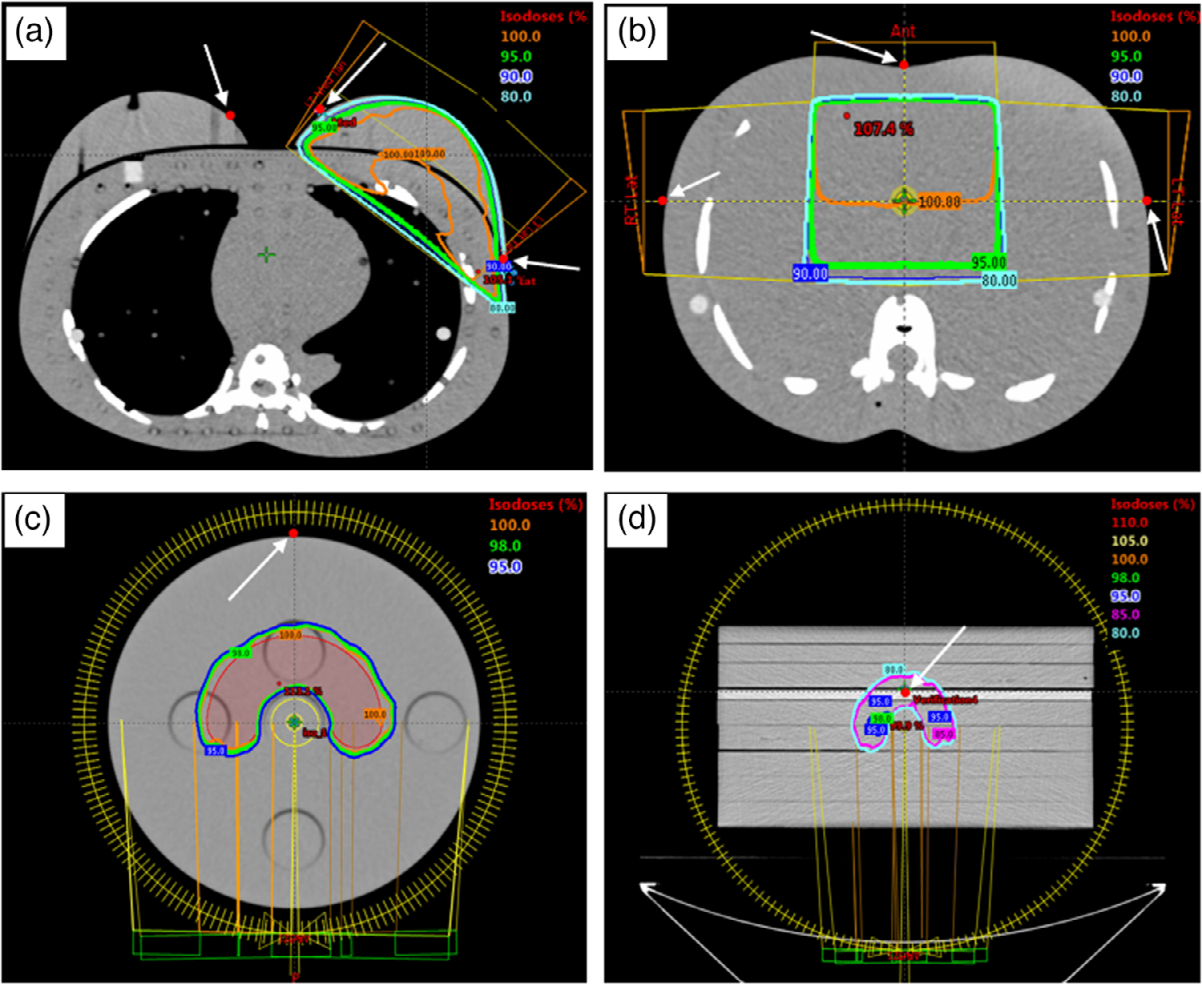
The beam arrangement and isodose distribution for (a) breast conformal radiotherapy, (b) abdomen conformal radiotherapy, (c) VMAT with CIRS IMRT Head and Neck phantom, (d) VMAT with solid water phantom. The position of the LuSy dosimeter is labelled as the red circle and indicated by the white arrows.

A breast and an abdomen treatment were selected for the conformal radiotherapy study. An anthropomorphic phantom (Radiology Support Devices, Inc., California, USA) was scanned with a 1 mm slice thickness and exported to the Eclipse TPS (Version 13.6, Varian Medical System, California, USA). The TPS has been commissioned and verified according to IAEA TRS430.^24^ Two tangential‐opposed fields were created for the breast plan, and a dynamic wedge was used to produce a homogenous dose distribution (Figure 3a). Two lateral‐opposed fields and one anterior field were arranged for the abdomen case. Both lateral fields had a dynamic wedge for homogenous dose distribution (Figure 3b). The treatment plans were created simulating real clinical cases, achieving 95% dose coverage to the target volume. Both plans used 6 MV photon beams with a 1 mm calculation grid size. The Anisotropic Analytical Algorithm (AAA) was used for dose calculation.

A C‐shaped test from the AAPM TG119 test suite was used for the IMRT and VMAT studies. A CIRS IMRT Head and Neck phantom (Computerised Imaging Reference Systems, Inc., Virginia, USA) was scanned and exported to the Eclipse TPS. The C‐shape target, the core structures, and the dosimeter volume were contoured, as shown in Figure 3c. Then, nine‐field beams were arranged at 40° intervals from the vertical plane for the IMRT plan. For VMAT, a two‐arc plan was created where each arc rotated 360° around the isocenter. The IMRT and VMAT cases were planned according to the planning goals provided in the AAPM test suite; 95% of the target volume received at least 5000 cGy, and less than 5% of the core volume received 2500 cGy. The fraction was set to 25 fractions.

Three spherical structures were created for SRS to mimic the multiple brain metastasis lesion. Each sphere is 2 cm in diameter and arranged in different planes. Then, a single‐arc VMAT plan was created with one isocenter where the prescribed dose was 2400 cGy in a single fraction. For IMRT, VMAT, and SRS plans, the energy used was 6 MV, and the calculation grid was set to 1 mm. The algorithm used for dose calculation was AAA.

### 2.4 Dose measurement

Dose measurement for conformal radiotherapy was made on the surface using the LuSy dosimeter, radiochromic film, and MO*Skin* dosimeter. For the breast irradiation, the dosimeter was positioned at three locations: 3 cm from the posterior field border of the medial‐tangential beam, 3 cm from the posterior field border of the lateral‐tangential beam, and on the medial side of the contra‐lateral breast (Figure 3a). For the abdomen irradiation, the dosimeters were positioned at three locations: the central axis of the anterior beam, the central axis of the right lateral beam, and the central axis of the left lateral beam (Figure 3b).

For IMRT, VMAT, and SRS, measurements were divided into two categories: in‐target and surface measurements. The created treatment plans were converted to the verification plans using the TPS for in‐target measurements. Solid water phantoms were used in the verification plan, and the dosimeter was positioned at a 5 cm depth from the phantom's surface (Figure 3d). For the LuSy dosimeter, the same fabricated acrylic phantom used for calibration was used to fix the position of the LuSy sensor (Figure 2a and b). Another 10 cm of solid water phantom was added as the backscatter. The phantom assembly was shifted so that the dosimeters were positioned within the target volume and homogenous dose distribution. For the SRS technique, one target was selected out of the three spherical structures and the dosimeter was shifted toward the center of the sphere. The LuSy dosimeter and the CC13 ionization chamber were used sequentially to measure the dose delivered using IMRT, VMAT, and SRS techniques.

For surface dose measurement, the LuSy dosimeter, radiochromic film, and MO*Skin* dosimeter were placed on the anterior surface of the CIRS IMRT Head and Neck phantom (Figure 3c). Regarding the SRS surface measurement, the same sphere that was selected for the in‐target measurement was used; however, the dosimeter was placed on the surface at the beam entry. Compared to in‐target irradiation, the actual plan was delivered instead of the verification plan and the plan prescription for IMRT and VMAT were reduced to 200 cGy. The prescription was reduced by changing the fraction to one, after the plan was optimized. Meanwhile, no prescription change was made to the SRS plan and the actual plan was delivered for the surface dose measurement.

The measured dose from all dosimeters was compared to the calculated dose by the TPS. The percentage deviation between the dose measured ($D_{\mathrm{meas}}$) and the dose predicted by TPS ($D_{\mathrm{calc}}$) was calculated using Equation (1);

(1) $$\Delta d\;\%=\frac{D_{\mathrm{meas}}-D_{\mathrm{calc}}}{D_{\mathrm{calc}}}\times 100$$

The stem effect was eliminated using the two‐fiber subtraction method.^6^, ^7^ The two‐fiber subtraction method used two optical fibers. One optical fiber was attached to the plastic scintillator, and the other, called the Cerenkov fiber, was not. To correct for the stem effect, the signal measured by the Cerenkov fiber was subtracted from the signal measured by the plastic scintillator fiber. Therefore, each measurement using the LuSy dosimeter was repeated, where the first measurement was conducted using the plastic scintillator fiber and the second measurement using the Cerenkov fiber. In addition, the measurement at each dosimeter position was made three times for reproducibility of the dose measurement.

## 3 RESULTS

**FIGURE 4:**
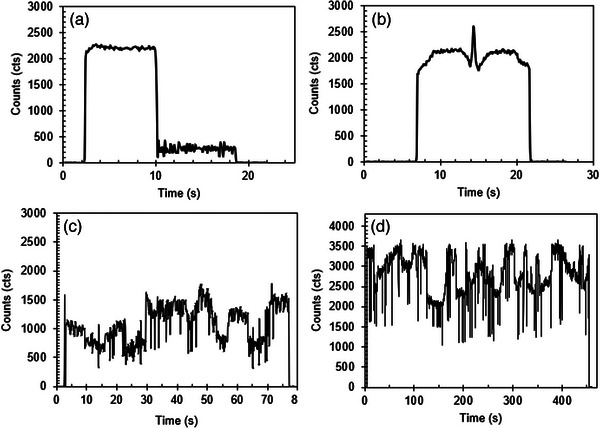
The raw signal measured using the LuSy dosimeter for (a) a single‐field wedged‐beam conformal radiotherapy, (b) a single IMRT field, (c) a single VMAT arc, and (d) a single SRS arc. The LuSy was set to readout at 100 ms time interval.

Figure 4 shows the raw signal measured for a single field by the LuSy dosimeter during conformal radiotherapy, IMRT, and VMAT. For conformal radiotherapy, the reduced signal at about the halfway point of the irradiation was caused by the dynamic wedge, which filters the dose to improve the dose uniformity within the treatment volume (Figure 4a). The dynamic wedge reduced the dose rate and moved the collimating jaw over the dosimeter to achieve the dose effect. IMRT technique used the sliding window MLC, resulting in the RL sensor being exposed to the radiation for a few seconds while the MLC modulates. From Figure 4b, a sudden spike was observed when the RL sensor was fully exposed to the ionizing radiation. In contrast to conformal radiotherapy and IMRT, the VMAT raw signal shows fluctuating signal across the radiation exposure, contributed by the dynamic MLC and the modulated dose rates constantly changing during the treatment delivery (Figure 4c). The raw signal for SRS is shown in Figure 4(d) where the signal amplitude is higher than conformal therapy, IMRT, and VMAT. This is due to the higher dose rate used for SRS treatment.

Breast irradiation resulted in a −5.5% mean deviation between the LuSy measurements and TPS dose calculated for the dosimeter positioned at the entrance of the medial and lateral tangential beams. Table 1 shows the absolute dose measured by all dosimeters and the dose calculated by the TPS. Comparing LuSy measurements with radiochromic film and MO*Skin* dosimeter resulted in the maximum dose difference of 8.9 and 44.1 cGy, respectively. The dosimeter positioned at the lateral tangential beam measured the maximum dose difference. Contra‐lateral breast dose measurement showed LuSy measuring 1.3 cGy lower than the TPS calculated dose. The capability of LuSy measuring the dose outside of the treatment field showed its sensitivity towards low doses.

**TABLE 1:** The dose measured at three dosimeter positions for breast and abdomen 3D conformal radiotherapy.

	Measured dose for breast (cGy)			Measured dose for abdomen (cGy)			Mean dose ratio of dosimeters from TPS		Mean dose ratio of dosimeters from film	
Dosimeter	Lateral	Medial	Contra‐lateral	Right	Centre	Left	Breast	Abdomen	Breast	Abdomen
Film	71.5 ± 2.0	75.5 ± 0.8	1.9 ± 0.5	55.9 ± 2.4	39.2 ± 2.0	54.1 ± 0.4	0.70	0.85	1.00	1.00
MO*Skin*	124.5 ± 2.0	118.6 ± 1.1	13.3 ± 0.9	58.7 ± 1.6	46.4 ± 2.5	64.7 ± 2.1	1.79	0.97	3.44	1.14
LuSy	80.4 ± 0.1	80.5 ± 1.0	4.0 ± 0.2	64.1 ± 0.5	41.8 ± 0.4	61.5 ± 0.3	0.88	0.95	1.43	1.12
TPS	85.7	84.5	5.3	67	45.2	64.3	1.00	1.00	1.70	1.18

The mean dose ratio is the ratio of the measured dose between each dosimeter to the TPS and Film.

Abdomen irradiation resulted in a −5.4% mean deviation between the LuSy dosimeter and the TPS calculated doses for all three dosimeter positions. Comparison of LuSy against radiochromic film resulted in a mean deviation of 11.6%, and LuSy showed a −9.9% maximum deviation against the MO*Skin* dosimeter.

For the advanced techniques, the mean deviation for the LuSy dosimeter in measuring the in‐target dose was −3.0% across the three treatment techniques. The mean deviation for the CC13 ionization chamber was 2.6% for the three treatment techniques. Furthermore, a comparison between LuSy and CC13 ionization chamber resulted in a −5.4% mean deviation. The dose measured by the dosimeters and the TPS calculated dose for each treatment technique is shown in Table 2.

**TABLE 2:** The in‐target dose was measured for advanced treatment techniques.

	Measured dose (cGy)			Mean dose ratio of dosimeters to TPS		
Dosimeter	IMRT	VMAT	SRS	IMRT	VMAT	SRS
CC13	184.9 ± 0.2	179.6 ± 0.1	2061.6 ± 1.7	1.03	1.02	1.03
LuSy	176.3 ± 0.5	167.8 ± 1.3	1957.6 ± 6.5	0.98	0.95	0.97
TPS	179.2	176.3	2009.2	1.00	1.00	1.00

The mean dose ratio is the ratio of measured dose between each dosimeter to the TPS.

Surface dose measurements for the advanced techniques show a greater deviation between the dosimeters and the TPS than in‐target dose measurements. Table 3 shows the absolute dose measured by all dosimeters and the dose calculated by the TPS. The mean deviation for the LuSy dosimeter against the TPS calculated dose is −1.8% across the three treatment techniques. The mean deviation for radiochromic film and MO*Skin* dosimeter against the TPS calculated dose was −19.4% and −19.3%, respectively. Comparing the LuSy dosimeter against radiochromic film and the MO*Skin* dosimeter for IMRT and VMAT resulted in a mean deviation of 6% and 4.8%, respectively. Additionally, SRS contributed the maximum dose difference between LuSy against radiochromic film and MOSkin dosimeter with 77.3 and 80.9 cGy, respectively.

**TABLE 3:** The surface dose was measured for advanced treatment techniques.

	Measured dose (cGy)			Mean dose ratio of dosimeters to TPS			Mean dose ratio of dosimeters to Film		
Dosimeter	IMRT	VMAT	SRS	IMRT	VMAT	SRS	IMRT	VMAT	SRS
Film	63.5 ± 1.4	66.3 ± 0.3	134.6 ± 2.1	0.90	0.78	0.74	1.00	1.00	1.00
MO*Skin*	65.9 ± 1.3	65.2 ± 1.4	131.0 ± 2.2	0.93	0.77	0.72	1.04	0.98	0.97
LuSy	69.0 ± 1.1	68.3 ± 0.9	211.9 ± 1.6	0.98	0.81	1.16	1.09	1.03	1.57
TPS	70.5	84.8	182.5	1.00	1.00	1.00	1.11	1.28	1.36

The mean dose ratio is the ratio of the measured dose between each dosimeter to the TPS and Film.

## 4 DISCUSSION

This study evaluated the feasibility of the LuSy dosimeter in measuring surface and in‐phantom absorbed dose for conformal radiotherapy, IMRT, VMAT, and SRS. It highlighted the use of SP101 plastic scintillator as the sensor, expanding the array of available scintillators for radiation measurements. Although the use of plastic scintillators in scintillation dosimetry is not new, the use of SP101 for dosimetry is unprecedented in the literature. Basic characterizations such as linearity, energy dependency, and dose rate dependency has been carried out previously.^20^, ^21^ This study focuses on the application of the LuSy dosimeter, where in‐target and surface‐dose measurements were conducted, and a comparison between LuSy measured dose against TPS predicted dose, ionization chamber, radiochromic film, or MO*Skin* measured dose was conducted.

Before performing dose measurements, the LuSy dosimeter must be calibrated to relate the measured light signal to the radiation dose. The calibration procedure is standard in radiotherapy, as other relative dosimeters such as semiconductor dosimeters, thermoluminescence dosimeters (TLDs), and optically stimulated luminescence dosimeters (OSLDs) also require a calibration process. From the calibration, 1 cGy will produce more than 200 counts, showing the sensitivity of LuSy. The calibration also yielded less than a 1% coefficient of variation, indicating that the LuSy dosimeter is highly reproducible.

This study adapted the two‐fiber subtraction method to eliminate the stem effect, resulting in a longer duration for the dose measurement procedure. For each measurement point, the exposure must be repeated for the plastic scintillator and Cerenkov fiber. In addition, adapting the two‐fiber subtraction method demands accuracy in the RL sensor positioning. For conformal radiotherapy, slight variation in the positioning of the plastic scintillator fiber and Cerenkov fiber does not affect the measured dose because of its static field size and homogeneous dose distribution; however, this became crucial when measuring IMRT, VMAT, and SRS techniques. These techniques require the MLC to be in smaller segments that modulate during radiation exposure. A slight offset between the position of the plastic scintillator fiber and the Cerenkov fiber will cause both fibers not measuring the same point. This will invalidate the subtraction method, as the point of measurement differs.

Other methods for stem effect elimination have been proposed by several groups,^2^ and those methods can be adapted to overcome the issue that arises from the two‐fiber subtraction method. Future work on the LuSy will involve exploring another method to eliminate the stem effect.

According to the IAEA Human Health Reports No. 8, the tolerance level for conformal radiotherapy is ± 5% for simple treatment and ± 7% for breast treatment.^25^ Therefore, the dose measured in this study for the breast treatment was within the tolerance level. Abdomen irradiation showed a −5.4% deviation, larger than the ± 5% recommended tolerance level. During the breast measurement, we observed that placing the RL sensor is vital because an offset will cause a different SSD for the tangential beam. The SSD directly affects the absorbed dose,^1^ causing higher deviation with the TPS.

The contra‐lateral breast was measured because it is an important organ at risk during breast radiotherapy. The dose to the contra‐lateral breast should be kept at less than 1 Gy to reduce the risk of second primary breast cancer in the contra‐lateral breast.^26^ The position of the contra‐lateral breast is outside of the treatment field; therefore, the measured dose is less than 10 cGy. The low doses are contributed by the photons and contaminants electrons scattered from the collimator head and scattered dose from the patient.

Dose measurement for IMRT, VMAT, and SRS showed a larger deviation from the TPS predicted dose. For these techniques, the in‐target measurement allows the user to select a dose point at a homogeneous dose distribution region and avoid the steep dose gradient regions. Selecting a dose point is critical to reducing the uncertainty of the positional error during the dose measurement.

In addition, using the fabricated acrylic phantom ensures the measurement position is reproducible for both treatment techniques. It is important for the stem effect correction method adapted where the RL sensor has to be inserted and removed multiple times. We observed an acceptable difference (−5.4%) when comparing the dose measurement between the LuSy dosimeter and the ionization chamber. However, the ionization chamber has its limitation by having a large sensitive volume which results in dose averaging. This limitation reduces the ionization chamber's accuracy in small‐field dosimetry.^27^ The advantage of using the LuSy dosimeter in this study is the high spatial resolution, allowing dose measurement within a small target volume such as the SRS treatment.

Surface dose measurement is challenging in radiotherapy due to (i) steep dose gradient regions, where the dose increases rapidly as the depth increases, and (ii) the lack of charge particle equilibrium (CPE) condition at the interface between two media, such as the air and the tissue.^28^ Furthermore, AAA algorithms have been reported to underestimate the surface dose by up to 14% of the prescription dose.^29^ In this study, the surface dose measurement resulted in a lower agreement with the TPS for the LuSy dosimeter, radiochromic film and the MO*Skin* dosimeter. The largest deviation was observed for the SRS technique (−28.2%), and based on the treatment plan, the dosimeters were in the steep dose gradient region. Therefore, positional error would highly impact the dose measured. Positional uncertainty affects the LuSy dosimeter and MO*Skin* dosimeter compared to film measurement. Film measurement is less susceptible to positional error due to its capability of measuring the dose in two‐dimensional (2D). For dose analysis, the user is capable to identify the measurement position by using a line profile. Such method is not possible for a one‐dimensional (1D) point dosimeter.

Apart from that, the discrepancies in measured dose between these dosimeters were contributed by the inherent differences between the LuSy dosimeter, radiochromic film, and the MO*Skin* dosimeter.

The inherent difference between the LuSy dosimeter and the MO*Skin* dosimeter is on the sensor tip. The geometry and dimension of the sensor tip for both dosimeters are significantly different. The RL sensor has a rod‐type design with a 1 mm diameter, and the MO*Skin* has a flat‐type design with a 0.35 mm thickness.^23^ These differences will affect the spatial resolution of the dosimeters and, thus, influence the dose measured in this study. Additionally, the effective point of measurement for each dosimeter varies from one another. The MO*Skin* was measuring at 0.07 mm WED, while the EBT3 was measuring at 0.16 mm WED.^30^ The LuSy dosimeter was expected to measure at a much deeper depth based on its physical dimensions and density. Therefore, the measurements by each dosimeter did not correspond to the same depth, which may contribute to the differences between the measured doses. While it is impossible to measure the surface dose at zero depth, taking into account of the dosimeter's physical dimension and the WEDs could provide valuable insights into the surface dose measurements. Approach such as surface dose correction can also be introduced for each dosimeter to predict the dose at the surface.^31^

Nevertheless, the surface dose for SRS treatment is less than 10% of the prescription dose. The significance of the deviation between the measured and calculated doses for the surface dose measurement should be evaluated before a clinical decision.

Several studies using plastic scintillators for IMRT and VMAT dose measurement have shown good agreement with the TPS calculated dose. Klein and Briere^32^ reported a 0.1% deviation for IMRT and a 0.7% deviation for VMAT. Huang and Qiao^33^ used the commercial plastic scintillator dosimeter, Extradin W1, to measure IMRT and VMAT doses. They reported a 0.27% deviation between the measured and calculated dose for IMRT treatment and −0.19% for VMAT treatment. However, both studies measured the dose within a phantom without measurement on the surface. Our results are comparable with these studies for similar measurement positions. To improve the dose measurement, a study by Snyder and Sullivan^34^ suggested calibrating the plastic scintillator dosimeter with a 4 × 4 cm^2^ field size for the VMAT dose measurement. This is because the techniques are delivered in smaller segments and calibrating at smaller field size match closer to the irradiation condition. For surface dose measurement, the AAA algorithm for the treatment planning system is known to have lower accuracy compared to Monte Carlo dose algorithm.^35^ Therefore, future study involving Monte Carlo algorithm, especially for the surface dose measurement, would be useful in evaluating the performance of the LuSy dosimeter. Apart from that, calibration at the surface of the phantom can also improve the dose measurement as the dosimetric condition at a depth of maximum dose differs from that of the surface.^28^ Future study on the dose measurement under bolus for treatment site such as breast could also be carried out.

One limitation of this study is the sample size. For each treatment technique, a single plan was created; therefore, the robustness of the LuSy dosimeter cannot be thoroughly evaluated. Nevertheless, the advantage of LuSy dosimeter is the sensitivity where it can measure the dose outside of the treatment field. Previous characterization also shows that the dosimeter is capable of measuring low‐energy photons, with a low dose.^20^ The readings are reproduceable, with less than 1% variation. LuSy dosimeter provides the dose information in real‐time, prompting immediate action for any dose correction during the patient's treatment.

## 5 CONCLUSION

We evaluated the feasibility of the LuSy dosimeter in measuring conformal radiotherapy, IMRT, VMAT, and SRS treatments. The measured dose was compared against the calculated dose by the TPS. The surface dose measured by LuSy dosimeter deviates −5.5% and −5.4% from the TPS for breast and abdomen treatments, respectively. In contrast, for advanced treatments such as IMRT, VMAT, and SRS, the LuSy dosimeter resulted in a −3.0% deviation for measurement inside the target volume. Surface dose measurements resulted in higher deviation than in‐target, where deviations for IMRT, VMAT, and SRS were −2.0%, −19.5%, and 16.1%, respectively. The larger dose discrepancies may be due to the high dose gradients and positional uncertainty associated with these advanced and small‐field techniques. Nevertheless, the need and value of performing in vivo dose measurements using appropriate dosimeters with a high spatial resolution and tissue equivalent properties are still valid, particularly as part of the comprehensive medical physics efforts towards improving patient safety in radiotherapy.

In the perspective of clinical application, considering the various factors that could lead to a larger uncertainty in dose measurements, including but not limited to factors such as non‐CPE conditions, tissue inhomogeneities, high‐dose gradients, positional uncertainties, TPS dose calculation algorithm, etc., it is crucial to understand the total compounding uncertainties associated with each type of dosimeters to provide a realistic expectation and confidence of the measured dose.

Though the sample size was minimal, the LuSy dosimeter shows promising results for clinical applications. The LuSy dosimeter has high spatial resolution and can perform real‐time dose measurements. These are the desirable characteristics of a dosimeter in ensuring patient safety during radiotherapy treatment.

## AUTHOR CONTRIBUTIONS

Janatul Madinah Wahabi: Investigation, methodology, data curation, data analysis, and writing the original manuscript; Jeannie Hsiu Ding Wong: Conceptualization, project administration, data validation, manuscript revision and supervision; Ghafour A. Mahdiraji: Resources, manuscript revision; Ngie Min Ung: Conceptualization, project administration, data validation, manuscript revision and supervision.

## CONFLICT OF INTEREST STATEMENT

No conflicts of interest.
